# A Rare Case of Cutaneous Leiomyosarcoma Arising From the External Auditory Canal

**DOI:** 10.7759/cureus.102558

**Published:** 2026-01-29

**Authors:** Jun Yamashita, Shinsuke Ide, Kei Kajihara, Takeshi Nakamura, Kuniyuki Takahashi

**Affiliations:** 1 Department of Otolaryngology - Head and Neck Surgery, Faculty of Medicine, University of Miyazaki, Miyazaki, JPN; 2 Department of Otolaryngology - Head and Neck Surgery, Faculty of Medicine, The University of Osaka, Suita, JPN; 3 Department of Otolaryngology - Head and Neck Surgery, Miyazaki Prefectural Miyazaki Hospital, Miyazaki, JPN

**Keywords:** cutaneous leiomyosarcoma, external auditory canal, fnclcc grade, head and neck sarcoma, treatment strategy

## Abstract

Leiomyosarcoma (LMS) is a malignant tumor that originates from smooth muscle. Cutaneous LMS is a distinct, superficial sarcoma that arises from dermal smooth muscle. It generally has a more favorable prognosis than subcutaneous or deep soft tissue LMS. Head and neck LMS accounts for a small proportion of all LMS cases, and primary tumors in the external auditory canal are extremely rare.

We report a case of cutaneous LMS arising from the external auditory canal. The patient was a 32-year-old woman. She presented with a two-month history of a left external auditory canal mass, hearing loss, and ear pain. Contrast-enhanced CT and MRI revealed a 17-mm tumor confined to the cartilaginous portion of the external auditory canal, with no obvious infiltration into the bony portion. Biopsy results showed features of a smooth muscle tumor, and the level of Ki-67 expression led to a diagnosis of LMS. We resected the tumor via a preauricular incision and a longitudinal incision of the external auditory canal, including the external auditory canal cartilage, the surrounding soft tissue, and part of the temporomandibular joint capsule. Histopathology revealed tumor cells with moderately atypical, spindle-shaped nuclei arranged in fasciculated patterns. Necrosis was observed at a rate of 30%-40%, and mitotic cells were observed at a rate of 5 per 10 high-power fields. This led to a diagnosis of Fédération Nationale des Centres de Lutte Contre le Cancer (FNCLCC) Grade 2 LMS. Tumor cells were noted near the resection margin, and the safety margin appeared inadequate. Postoperative epithelialization of the external auditory canal was favorable, and no recurrence was detected on contrast-enhanced CT at five months.

Although there is no established treatment strategy for head and neck soft tissue sarcomas, complete resection with adequate margins is essential. However, achieving adequate surgical margins is challenging due to functional and cosmetic considerations in the head and neck region. As in this case, small, brownish LMS are clinically considered to be cutaneous LMS, which generally have a relatively good prognosis. Close follow-up is required in cases involving resection close to the margin, and adjuvant therapies such as radical resection or radiation should be considered if recurrence occurs. A treatment strategy tailored to the tumor type and resection margin is necessary for primary LMS of the head and neck region.

## Introduction

Leiomyosarcomas (LMS) are malignant tumors of smooth muscle origin and are common histological subtypes of soft tissue sarcoma [1]. Superficial LMS on the skin surface is classified as cutaneous or subcutaneous, as it can also develop in deep tissues. Cutaneous LMS are thought to originate from the arrector pili muscles and usually have an excellent prognosis, whereas subcutaneous LMS originate from vascular smooth muscle and are more likely to recur and metastasize [2-4]. Cutaneous and subcutaneous LMS have a distinctly different clinicopathological spectrum from deep soft-tissue LMS.

Most LMS originate in the retroperitoneum, uterus, and extremities [1]. Only 4.1% occur in the head and neck region [5], and rarely in the external auditory canal [6-8]. A staging system and guidelines have been established for treating soft tissue tumors of the extremities and trunk. However, guidelines to treat primary soft tissue sarcomas of the head and neck are lacking, and surgical resection with a sufficient margin is generally recommended. However, due to the specific nature of the head and neck region, treatment strategies must also consider postoperative functional impairment and cosmetic outcomes. In particular, tumors in the external auditory canal can affect hearing, balance, facial movement, and dental occlusal function, due to their proximity to important surrounding organs. In this report, we present a case of a cutaneous LMS that originated in the external auditory canal and review the literature.

## Case presentation

The patient was a 32-year-old woman who had suffered from a mass in her left external auditory canal and hearing difficulties for two months. She subsequently developed ear pain and visited our hospital.

**Figure 1:**
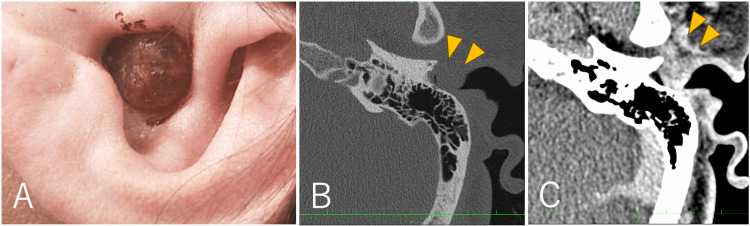
Preoperative photograph and CT images (A) Preoperative photograph of the left auricle showing a brownish mass occupying the entrance of the external auditory canal. (B) Temporal bone CT revealed a 17-mm mass in the cartilaginous portion of the external auditory canal (arrowhead) without obvious invasion into the bony portion. (C) Contrast-enhanced CT demonstrated uniform enhancement of the mass with partial enhancement of the temporomandibular joint capsule (arrowhead).

**Figure 2:**
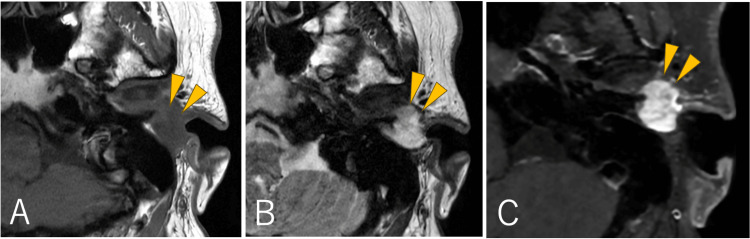
Preoperative MRI Preoperative MRI showing tumor hypointensity (arrowhead) on T1-weighted imaging (A), hyperintensity on T2-weighted imaging (arrowhead) (B), and heterogeneous enhancement on gadolinium-enhanced imaging (arrowhead) (C).

The brownish mass with a smooth surface was located at the entrance to the left external auditory canal and was connected to the anterior wall (Figure 1A). The tumor completely obstructed the external auditory canal, and the tympanic membrane could not be visualized. The patient had mixed hearing loss, with an air-bone gap of up to 60 dB and elevated bone conduction thresholds at frequencies of 1 and 2 kHz. Temporal bone CT revealed a 17 mm mass in the cartilaginous portion of the external auditory canal, with no obvious invasion into the bony portion (Figure 1B). Contrast-enhanced CT showed that the entire mass had enhanced, with the temporomandibular joint capsule partially enhanced (Figure 1C). MRI revealed a T1-hypointense and T2-hyperintense mass, with a slightly heterogeneous enhancement pattern within the mass (Figures 2A-2C).

Biopsy specimens demonstrated proliferating spindle-shaped tumor cells, with areas of necrosis and a few mitotic figures. Immunohistochemical analysis showed positivity for vimentin, alpha-smooth muscle actin (α-SMA), and desmin, and negativity for S-100, epithelial membrane antigen (EMA), and CD34. These results indicate that the tumor is of smooth muscle origin. Additionally, the Ki-67 labeling index was high, at approximately 30%, suggesting the possibility of malignancy.

**Figure 3:**
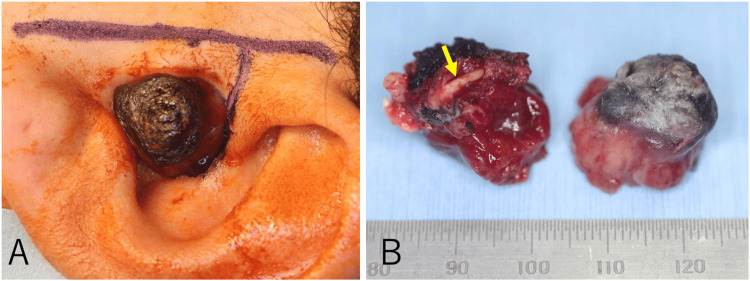
Skin incision and resected specimen photographs (A) A skin incision was made parallel to the auricle in the preauricular region. A vertical incision was also made in the external auditory canal between the tragus and crus helix. (B) The tumor was divided into two sections and resected along with part of the cartilaginous external auditory canal (yellow arrow), the surrounding soft tissue, and part of the temporomandibular joint capsule. Left: cross-section of the cut tumor specimen and Right: lateral surface of the tumor.

Due to the potential malignancy and rapid growth of the mass, excision under general anesthesia was performed promptly. Since the lesion was confined to the cartilaginous portion of the external auditory canal, reconstruction using a flap was not planned, and local excision was deemed feasible. First, a skin incision was made parallel to the auricle in the preauricular region. Then, a vertical incision was made into the external auditory canal, between the tragus and crus helix (Figure 3A). We initially aimed to resect the tumor and the surrounding normal tissue en bloc, but the deep portion of the external auditory canal could not be visualized. Therefore, we divided the tumor into two parts to safely dissect the deeper portion. After tumor resection, the tympanic membrane and bony external auditory canal appeared normal. As shown in the preoperative imaging, the tumor was attached to the temporomandibular joint capsule. The tumor was resected along with part of the cartilaginous external auditory canal, the surrounding soft tissue, and part of the temporomandibular joint capsule (Figure 3B). Since the tumor showed no signs of invasion, and for cosmetic reasons, the tip of the tragus was preserved. The skin defect inside the auditory canal was left as an open wound with a raw surface. The superficial location in the cartilaginous external auditory canal, brownish color, and the absence of deep soft tissue or bony invasion supported the notion that the tumor was primary cutaneous LMS.

**Figure 4:**
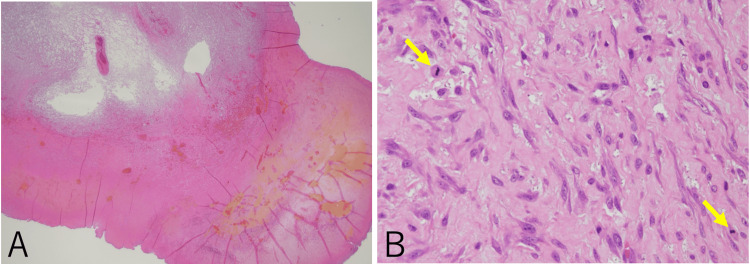
Pathological findings (A) Low-power field image magnified ×12.5 of a hematoxylin and eosin-stained specimen. Tumor necrosis is visible in the lower right region. (B) High-power field image magnified ×400. Mitotic cells were sporadically observed (yellow arrows highlighting the mitotic figure).

Histopathology revealed that tumor necrosis was present in 30%-40% of the specimens (Figure 4A). The tumor cells, which had moderately atypical, spindle-shaped nuclei, were arranged in fasciculated patterns. Mitotic cells were sporadically observed at a rate of 5 per 10 high-power fields (Figure 4B). The MIB-1 index, synonymous with Ki-67, was high, at 10.8% (339 positive cells out of 3,140 total cells). The discrepancy in the Ki-67/MIB-1 index between the biopsy and the resected specimen was likely due to tumor heterogeneity, sampling variation (site and timing), and the influence of progressive necrosis in the larger tumor. Immunohistochemistry showed the same pattern at the time of biopsy. Based on these findings, the diagnosis was confirmed as Grade 2 LMS, according to the Fédération Nationale des Centres de Lutte Contre le Cancer (FNCLCC) Histologic Grading System (Table 1) [9]. Evaluating the margin by microscopy was challenging because the tumor cells exhibited weak atypia, low cell density, and indistinct borders, along with bipolar cautery damage. These findings made it difficult to determine whether the cells within the margin were tumor cells or normal stromal cells. Therefore, after consulting with the pathologist, the deep margin was interpreted as a possible R1 resection. 

**Table 1:** Fédération Nationale Des Centres De Lutte Contre Le Cancer (FNCLCC) Histologic Grading System for soft tissue sarcomas Credit: Modified from Coindre [9]

Parameter	Criteria	Score
Tumor differentiation	Well-differentiated sarcoma resembling normal mesenchymal tissue	1
	Sarcoma for which histologic typing is certain	2
	Embryonal and undifferentiated sarcomas, sarcomas of doubtful type, synovial sarcomas, osteosarcomas, primitive neuroectodermal tumor (PNET)	3
Mitotic count (per 10 HPF)	0-9 mitoses/10 HPF	1
	10-19 mitoses/10 HPF	2
	≥20 mitoses/10 HPF	3
Tumor necrosis	No necrosis	0
	<50% necrosis	1
	≥50% necrosis	2
Total score	Sum of differentiation + mitotic count + necrosis	-
Final histologic grade	Total score	
	2-3 → Grade 1	
	4-5 → Grade 2	
	6-8 → Grade 3	

**Figure 5:**
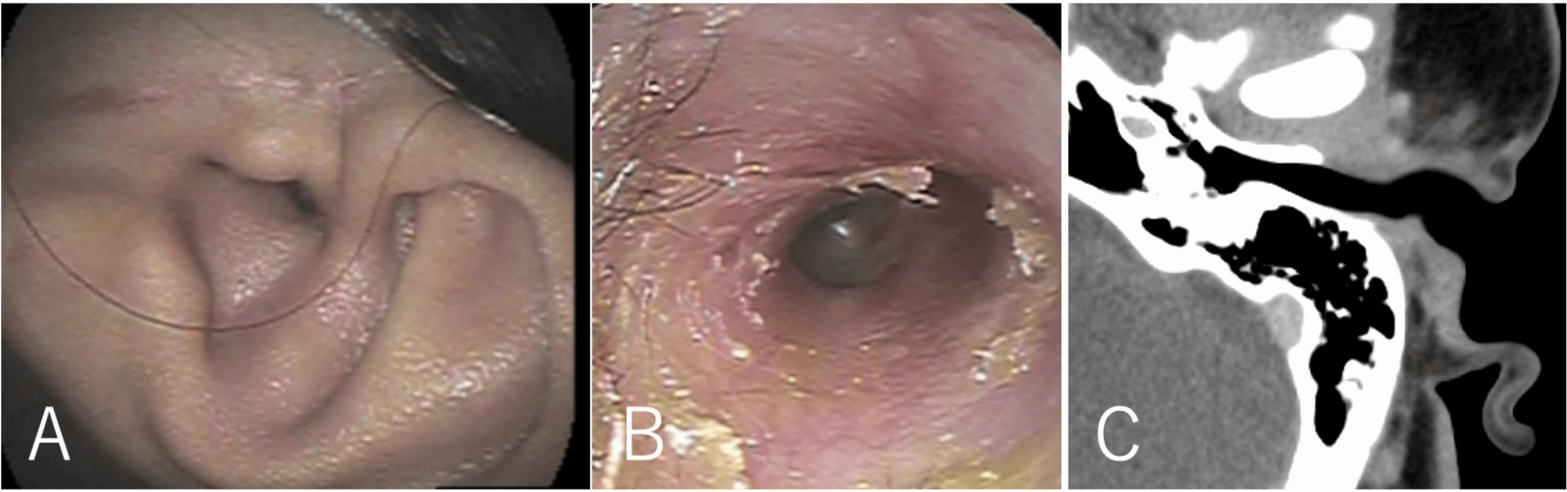
Postoperative findings Postoperative photographs of the left auricle (A) and external auditory canal (B). (C) Contrast-enhanced CT image taken five months postoperatively. The tumor has disappeared, and the external auditory canal is well-epithelized, with the tympanic membrane visible. Contrast-enhanced CT showed no sign of recurrence.

There were no obvious postoperative complications, such as balance disorders, facial nerve palsy, or dental occlusal discrepancies, and subjective hearing loss was resolved. The external auditory canal underwent normal epithelialization (Figures 5A, 5B), and there were no signs of local recurrence. A contrast-enhanced CT scan performed five months postoperatively showed no evidence of recurrence (Figure 5C).

## Discussion

Soft tissue sarcomas are extremely rare, accounting for less than 1% of all malignant tumors. They are considered one of the rarer types of malignant tumors, with an incidence rate of approximately 2.9 to 5.6 cases per 100,000 people per year [10,11]. The frequency of histological types differs between children and adults. In adults, gastrointestinal stromal tumors (GISTs) are the most common mesenchymal tumors of the gastrointestinal tract [12]. Among non-GIST soft tissue sarcomas, LMS is a prevalent histologic subtype that accounts for 10%-20% of all sarcomas [1,5].

LMS is classified as either superficial, which appears on the skin, or deep, which originates within the body. Specifically for superficial LMS, there are two types: cutaneous and subcutaneous. Cutaneous LMS originate in dermally located hair follicle, dartos, or areolar muscles, whereas subcutaneous LMS originate in the vascular smooth muscle of subcutaneous adipose tissue. Cutaneous LMS are typically smaller than subcutaneous LMS. They are usually found on the lower extremities, trunk, or scalp, and present as painful, erythematous to brownish nodules [3,13]. The tumor in our patient was diagnosed as cutaneous LMS based on its clinical features. The prognosis of cutaneous LMS is good [2-4]; thus, it should be differentiated from subcutaneous types to predict prognosis and ensure appropriate treatment strategies. In the external auditory canal, the presumed primary sites of origin are the arrector pili muscle and vascular smooth muscle within the skin of the cartilaginous portion. Therefore, most of these tumors are thought to be superficial LMS. Nonetheless, LMS occurring in the external auditory canal has been reported in only a few cases and is extremely rare [6-8].

Various tumorous lesions occur in the external auditory canal, but their precise incidence rates and pathological classifications are unknown. Among benign tumors, nevi, osteomas, and papillomas are the most common [14,15]. Exostoses and cholesteatomas, which are not tumors, are also frequently observed as masses in the external auditory canal [14]. Malignant tumors of the external auditory canal occur at a rate of one to six cases per million people per year, accounting for approximately 0.2% of all head and neck malignancies [16]. Pathologically, squamous cell carcinoma predominates. In the current case, the soft, smooth surface, along with the absence of bone destruction, initially suggested a benign tumor. However, the presence of pain and rapid growth necessitated consideration of malignancy. The tumor’s location made biopsy easy, enabling early diagnosis of external auditory canal LMS.

Soft tissue sarcomas are classified into three grades according to the FNCLCC system, which considers tumor differentiation, mitotic count, and tumor necrosis [9]. In this case, the tumor differentiation score was 2, the mitotic count score was 1, and the tumor necrosis score was 1. These scores correspond to a histologic grade 2. For tumors of the extremities and trunk, the American Joint Committee on Cancer (AJCC) staging system employs size, lymph node involvement, and distant metastasis, in addition to the FNCLCC classification, to determine treatment strategies [17]. However, no established staging system exists for primary soft tissue sarcomas of the head and neck. Generally, surgical resection with a sufficient margin is recommended for soft tissue sarcomas. Due to the specific nature of the head and neck region, however, treatment strategies must also consider postoperative functional impairment and cosmetic outcomes [18]. Surgical resection is classified as R0 (complete resection with negative margins), R1 (incomplete resection with microscopically positive margins), or R2 (incomplete resection with macroscopically positive margins). The prognosis for soft tissue sarcomas of the head and neck is poorer when associated with a higher pathological grade according to the FNCLCC classification [19,20], poorer surgical resection margins [21,22], a tumor size exceeding 5 cm [19,20], and older age [22]. For LMS specifically, cutaneous LMS have a lower recurrence and metastasis rate and a better prognosis than subcutaneous LMS [2,3,23]. Consequently, some recommend resection with a 1 cm safety margin for cutaneous LMS and a 2 cm margin for subcutaneous LMS [3]. Adequate margins cannot be defined only by linear distance in the external auditory canal. They must also be evaluated relative to surrounding tissue planes, including the skin and cartilage of the canal, the underlying temporal bone, the temporomandibular joint capsule, and the parotid/facial nerve region. Extending the resection further into these regions would result in significant functional and cosmetic impairment. Therefore, we decided on a very close, deep margin and compensated for it by cautious postoperative surveillance.

Regarding postoperative adjuvant therapy, some reports indicate that radiation therapy does not improve survival [23]; however, others suggest that it reduces the risk of recurrence in R1 and R2 resection cases [18]. Similarly, there are reports indicating that chemotherapy does not clearly improve survival [18]. Therefore, the gold standard treatment for head and neck LMS is radical resection, with adjuvant therapy selected based on the extent of resection. We considered that the tumor in our patient was cutaneous LMS based on its size and clinical features. Although the prognosis of this type of tumor is generally good, we could not achieve a definite R0 resection with an adequate safety margin. Consequently, close follow-up is warranted. If recurrence occurs, extended resection and adjuvant therapy should be considered. Although no recurrence was observed nine months after surgery, longer surveillance is necessary. Current literature on cutaneous LMS indicates that clinical examinations should be conducted at least every six months for the first two years and annually thereafter [3]. Only a few LMS in the external auditory canal have been reported [6-8], and detailed information about tumor size, surgical methods, or resected margins was scant. Recurrences have not appeared during short-term follow-up, but long-term outcomes have not been reported. Our case contributes to the limited literature regarding LMS in the external auditory canal. It highlights the difficulty of achieving an adequate surgical margin in an anatomically constrained site and emphasizes the need for careful long-term surveillance.

## Conclusions

We present a rare case of a cutaneous LMS arising from the external auditory canal. Soft tissue sarcomas in the head and neck region are extremely rare, and definitive treatment strategies remain unclear. However, it is important to distinguish between the types of LMS, especially cutaneous and subcutaneous, because their treatment strategies, follow-up approaches, and prognoses differ. While complete resection with adequate margins is the standard treatment for LMS, treatment strategies in the head and neck region must consider postoperative functional impairment and cosmetic outcomes. Therefore, we emphasize the difficulty of achieving an adequate surgical margin in this anatomically constrained site and the need for careful long-term surveillance.
